# Rehabilitation of Patient with Acquired Maxillary Defect, using a Closed Hollow Bulb Obturator

**DOI:** 10.4103/0973-1075.78453

**Published:** 2011

**Authors:** Abhilasha S Bhasin, Virendra Singh, Sneha S Mantri

**Affiliations:** Department of Prosthodontics, Hitkarini Dental College and Hospital, Jabalpur, Madhya Pradesh, India; 1 Vyas Dental College and Hospital, Jodhpur, Rajasthan, India

**Keywords:** Definitive closed hollow bulb obturator, Hemimaxillectomy, Maxillectomy, Palliative care, Retention, Stability

## Abstract

Palliative care means providing support and care for patients with life-threatening or debilitating illness so that they can live their life as comfortably as possible. The fact that cure is no longer a reality does not mean that care cannot be made available. Partial maxillectomy defect presents a prosthodontic challenge in terms of re-establishing oronasal separation. Such defect has direct effect on cosmetic, function and psychology of the patient. This article describes step by step clinical and laboratory procedures involved in the rehabilitation of a hemimaxillectomy patient, using a definitive closed hollow bulb obturator, which improved his physical, emotional, functional, social and spiritual needs.

## INTRODUCTION

Mouth and oropharyngeal cancers can be treated with surgery, radiation, chemotherapy or biological therapy. Treatment is decided according to the type of cancer, stage and grade of cancer, the impact of treatment on patient’s speech, chewing, swallowing and general health and fitness. Any alternative treatment which helps to improve the quality of life of a patient is palliative care. Acquired maxillofacial defects are those that are created by other than congenital/developmental influences. These are manmade defects more often related to surgical intervention for the elimination of disease process, trauma resulting in significant alteration of the normal anatomic features of the oral and facial structures. Maxillectomy defects result in the formation of an opening between the oral cavity and the antrum and/or the nasopharynx, creating problems with speech, mastication, swallowing and impaired facial esthetics.[1]

Obturator is that component of a prosthesis which fits into and closes a defect within the oral cavity or other body defect.[2] In edentulous patients, support, stability and retention of obturating removable prosthesis depends on the remaining hard and soft tissues.[3] The larger the defect, greater the loss of the mucogingival support.[4,5] This case report describes the clinical and laboratory procedures involved in the rehabilitation of a patient with hemimaxillectomy defect, using a closed bulb hollow obturator.

## CASE REPORT

A 62-year-old patient reported to the Department of Prosthodontics for replacement of his maxillary and mandibular teeth. He had complains of hypernasality of voice, regurgitation of food in the nasal cavity, and difficulty in eating and speech. Dental history revealed that the patient had undergone maxillectomy 3 years back for myxomatous growth in palate with septal perforation. After surgery, maxillary posterior teeth were extracted. The patient had been wearing removable partial denture with a hollow bulb obturator since then. One month back, the patient underwent extraction of his remaining maxillary and mandibular teeth, so the previous prosthesis could not be used.

Extraoral examination revealed collapsed left maxillary and nasal region. His intraoral examination revealed completely edentulous maxillary and mandibular arches with healed socket. Palatal defect extended from the premaxillary region in the midpalatal portion, maintaining the continuity of the alveolar ridge anteriorly, extending posteriorly and involving the soft palate and uvula. Roof of the nasal cavity and the superior wall of the nasopharynx were visible through the defect. Our treatment objective was to provide prosthesis to obturate the defect to improve speech, deglutition and mastication, to restore facial contour and to replace the lost teeth. Maxillary and mandibular complete denture with maxillary definitive closed hollow bulb obturator was planned for the patient.

## PROCEDURE

**Figure 1:**
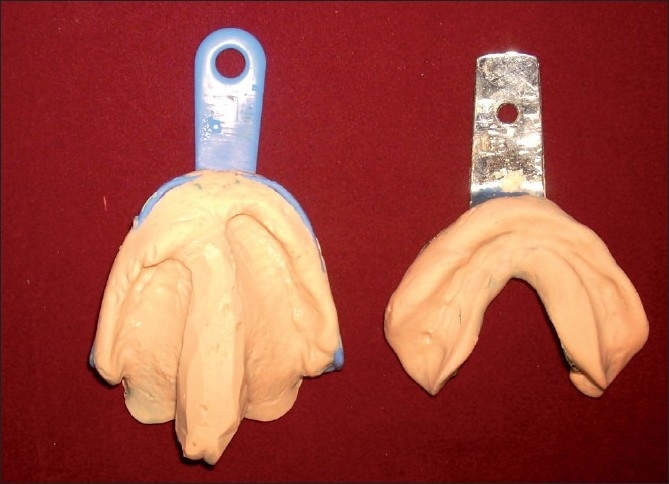
Primary impressions of maxillary arch with the palatal defect and the mandibular arch recorded using Alginate impression material

**Figure 2:**
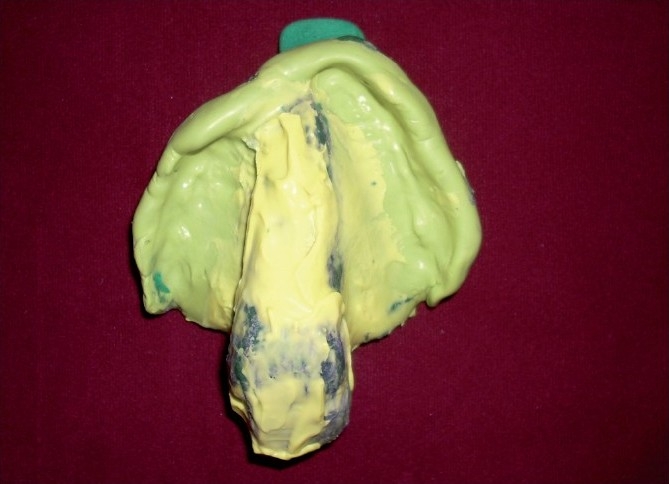
Final impression of maxillary arch

Maxillary and mandibular primary impressions were made using alginate impression material [Figure 1]. Undercuts in the maxillary defect area were blocked using wet cotton. Primary cast obtained was used for fabricating custom tray for final impression using autopolymerizing resin. Border molding was done to record the soft tissue surrounding the defect using low fusing impression compound. Details of the defect area were recorded using light body addition silicone elastomer (3M ESPE Express STD, Germany) and the wash impression was completed using medium body addition silicone elastomer [Figure 2]. Final impression of lower arch was completed using zinc oxide eugenol impression paste. Master cast obtained was duplicated. The duplicated cast was used to fabricate the bulb of the obturator. The defect area in the original cast was blocked out using plaster. On this cast, denture base and wax rims were prepared to record jaw relations, followed by try in of wax-up dentures.

**Figure 3:**
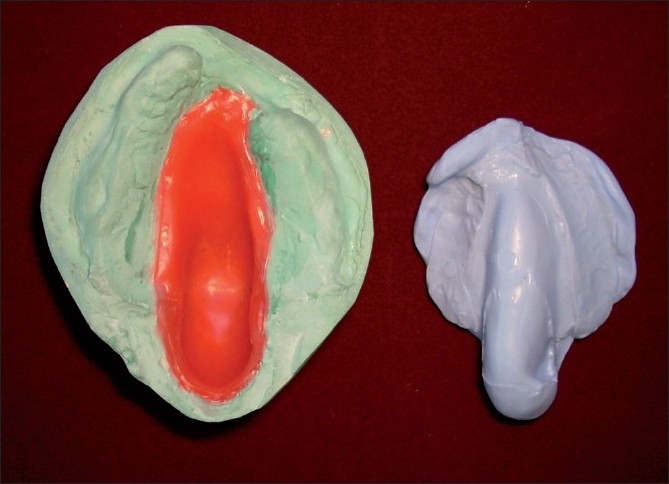
Duplicate master cast with a double layer of modeling wax lining adapted in the defect area. Over this wax lining, a silicone putty index was adapted to hollow the obturator bulb

**Figure 4:**
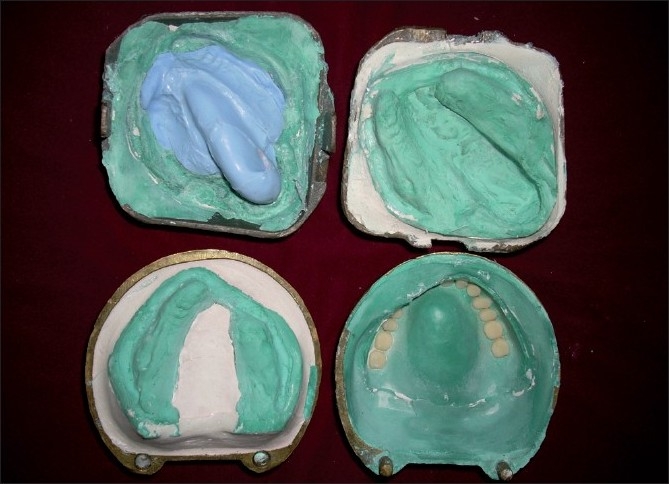
Two separate denture flasks used for acrylisation of the hollow obturator. The waxed up maxillary denture was flasked in first flask to obtain mold for maxillary denture. The second flask was used to pack the duplicate cast with the putty index for the hollow bulb

**Figure 5:**
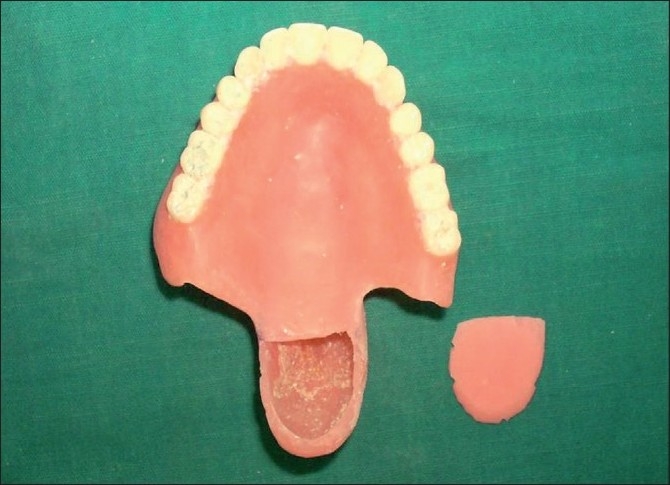
The hollow bulb and a lid for the bulb were prepared separately. Both the bulb to the denture base and the lid to the bulb were attached using autopolymerizing acrylic resin

**Figure 6:**
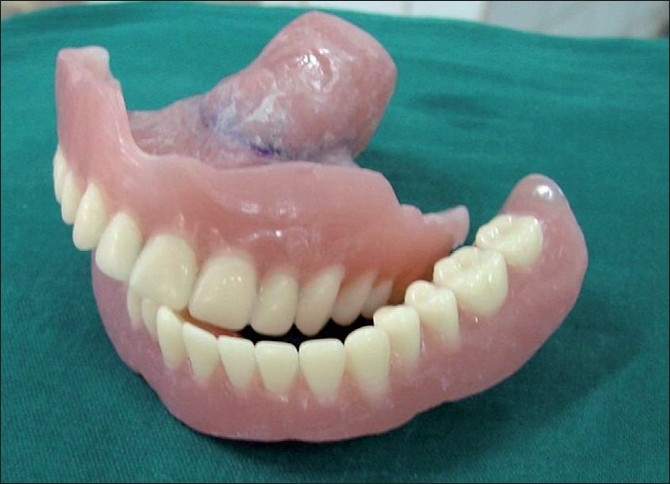
Finished and polished maxillary denture with the closed hollow bulb obturator and the mandibular denture

**Figure 7:**
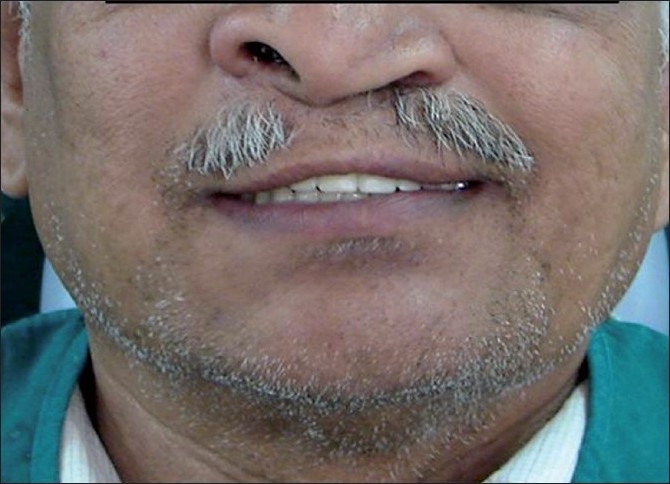
Dentures inserted in the patient’s mouth

On the duplicate cast, a double layer of modeling wax was adapted in the defect area. Over this wax lining, a silicone putty index was adapted. This putty index was used to hollow the obturator bulb [Figure 3]. Two separate denture flasks were used for acrylization of the hollow obturator. The waxed up maxillary denture was flasked in one flask. Dewaxing, packing with heat polymerized acrylic resin and curing were done to obtain maxillary denture [Figure 4]. The second flask was used to pack the duplicate cast with the putty index. The hollow bulb was fabricated separately in the second flask, was attached to the denture base using autopolymerizing acrylic resin and was floated in water to ensure complete seal. A lid for the bulb was prepared and attached to the open end of the bulb using autopolymerizing acrylic resin [Figures 5, 6]. The mandibular denture was fabricated using conventional technique. Both the dentures were finished, polished and inserted in the patient’s mouth [Figure 7]. Post insertion results showed improvement in speech, mastication, swallowing and facial esthetics. The patient was satisfied with the prosthesis in the recall checkups. Hygiene maintenance of the prosthesis was emphasized by home protocol instituted by the patient.

## DISCUSSION

All head and neck cancer patients should be under the care of a multidisciplinary team (M D T). This is a team of health professionals who work together to decide on the best way forward for each patient. The MDT includes oncologist, specialist nurse, dietician, prosthodontist and speech therapist. Care needs are dictated by medical and biological factors, demographics and psychological factors. Thus, care needs are unique to each patient. However, the delivery of care for those in transitions and for those in need of assessment, planning and ongoing management can be quiet challenging. Surgical resection of maxilla results in communication between oral and nasal cavity affecting deglutition, speech and facial esthetics. Along with these functional impairments, it can also be psychologically debilitating to the patient. Obturator prosthesis is commonly used for rehabilitating such patients. In the 18^th^century, a French surgeon, Ambroise Pare, described Button obturator made up of “cuff link” or “sponge”. In 1820, Delabarre used steel metal plate with wired metal bands clamped on teeth. This was the first artificial velum designed. In 1953, Ackerman fabricated hollow obturator prosthesis. Recent investigations have confirmed the effect of obturator prosthesis in terms of speech, swallowing and appearance.[6] In 1978, Dr. Mohammed Aramany presented a system of classification based on the relationship of the defect to the remaining teeth and the frequency of occurrence.[7] The defect in this case corresponded to the Aramany’s Class III occurring in the central portion of the hard palate and involving part of soft palate. Prosthetic obturation was the treatment planned for the patient with maxillectomy defect presented in this article. Since the maxillectomy defect was large, the retention and stability of the obturator was enhanced by making the obturator portion of the denture hollow. It improves the cantilever mechanics of suspension and avoids overtaxing of the remaining supporting structures.[8] For improving stability, maximum extension of the prosthesis in all lateral directions was provided so that the defect itself would enhance stability of the prosthesis.[9]

## CONCLUSION

Rehabilitation of an acquired maxillary defect in a patient, using definitive closed hollow bulb obturator, took care of the different domains of care, giving the patient an opportunity to live life as close to normal as possible. Teamwork enables the broader needs of patients and families to be addressed.
